# RELATIONSHIP SATISFACTION MEDIATES LINKS BETWEEN OLDER ADULTS’ SOCIAL NETWORKS AND DEPRESSION

**DOI:** 10.1093/geroni/igae098.2430

**Published:** 2024-12-31

**Authors:** Debarati Kole, Bryce Van Vleet, Heather Fuller

**Affiliations:** North Dakota State University, Fargo, North Dakota, United States; North Dakota State University, Fargo, North Dakota, United States; North Dakota State University, Fargo, North Dakota, United States

## Abstract

Social ties have a significant positive effect on older adults’ psychological well-being. However, limited research has examined the role of relationship satisfaction for the interplay between social ties and psychological well-being. The current study explores if relationship satisfaction mediates the relationship between social network characteristics and depressive symptoms among older adults. The data are from the first three waves of the Social Integration and Aging Study, a community-based, longitudinal study of older adults (aged 60+) in the Upper Midwest. Simple longitudinal structural equation modeling was used to analyze mediation models with baseline demographic variables (Age, Gender, and Education included as covariates. Three network variables (network size, mean proximity, and mean years of connection) were selected from Wave 1 (2013). A four-item relationship satisfaction construct (satisfaction from close family, extended family, friends, and neighbors) from Wave 2 (2015) was used as the mediator variable. The 10-item Geriatric Depression Scale, at Wave 3 (2017) was used as the outcome variable. Relationship satisfaction was found to mediate the effects of network size (paths: a=.022 p=.008; b=-.538 p<.001; c’=-.026 p=.239) and mean network proximity (paths: a=.361 p=.048; b=-.548 p<.001; c’=-.523 p=.237) on depressive symptoms over time. However, relationship satisfaction did not mediate the relationship between years of connection and depressive symptoms. These findings suggest qualitative aspects of social connections more greatly impact older adults’ psychological well-being over time as compared to quantitative social network characteristics. Implications for quality of connections and sense of belongingness will be discussed.

